# Genetics of kidney stones and the role of genetic testing in prevention: a guide for urologists

**DOI:** 10.3389/fmed.2025.1631281

**Published:** 2025-07-25

**Authors:** Francesco Pintus, Noemi Giordano, Daniela Francesca Giachino, Giorgia Mandrile

**Affiliations:** ^1^Department of Clinical and Biological Sciences, University of Turin, Orbassano, Italy; ^2^Genetic Unit and Thalassemia Centre, San Luigi University Hospital, Orbassano, Italy

**Keywords:** kidney stone, genetic testing, polygenic risk score, genetic predisposition, hereditary kidney stone disease

## Abstract

Kidney Stone Disease (KSD) has a high prevalence (approximately 10%) and high recurrence risk: almost half of stone former patients will experience recurrence within 5–10 years. To date, KSD is managed mostly surgically with a heavy burden on the healthcare system and numerous invasive procedures for the patients. In the past years a genetic basis in KSD has been increasingly recognized, with a heritability rate reaching 50%. Through Genome-Wide Association Studies (GWAS) and Next-Generation Sequencing (NGS) several genetic causes of recurrent nephrolithiasis have been untangled, paving the way to new therapies and prevention strategies, through precision medicine-based approaches. Many loci with more than 200 unique genes have been associated with KSD susceptibility thanks to GWAS, even though the development of a polygenic risk score is still in progress. Moreover, today, about 40 genes linked to monogenic disease that are involved in kidney stones have been identified, leading to a precise diagnosis in cases that were previously considered idiopathic. Despite these advancements, genetic testing in kidney stone formers remains underutilized and inconsistently available. The absence of clear diagnostic guidelines, standardization, and widespread awareness, combined with lack of perceived benefit, has left the decision to test largely at the discretion of individual physicians. This paper reviews the updated evidences in KSD genetics and suggest a diagnostic algorithm aimed to increase the diagnostic rate of genetic stones, allowing a personalized treatment and, in turn, a higher disease-free survival for the patients and a more efficient allocation of resources, analyzing the cost-effectiveness of genetic testing in urolithiasis. Besides, it will provide a further look to promising prospects in the field of prevention methods for kidney stones.

## 1 Introduction

Kidney Stone Disease (KSD) prevalence is currently rising worldwide reaching approximately 10% in the US, Europe, and Asia (1). Half of stone formers will experience recurrence within 5–10 years and 75% within 20 years, this leading to an increased risk of chronic kidney disease (CKD) and kidney failure (2).

Calcium stones are reported in 94% of first-time stone former patients, followed by uric acid (4.8% of first-time stone formers), while brushite and struvite compositions are rare (0.9%) and cystine very rare (0.1%). Among common calcium stones, calcium oxalate (76%) is the most common composition (3).

Stone formation may have an underlying genetic base which can therefore explain a high risk of recurrence and thus needs to be investigated for better prevention and treatment.

Indeed, in the last years has emerged that KSD has an important genetic basis with a heritability approximately of 50%, as observed in twin studies (46% for female twins and 62% for male twins) (4, 5). The pathogenesis of KSD remains unclear in many cases, but it is likely due to both genetic and environmental factors. In the past decades, numerous Genome-Wide Association Studies (GWAS) and New Generation Sequencing (NGS) studies demonstrated a genetic cause in several cohort of nephrolithiasis patients, paving the way for new possible opportunities of therapy and prevention through precision medicine-based strategies (2, 6–9), likely reducing the burden on the healthcare system and offering new treatments besides surgical management of KSD (10, 11).

## 2 Guidelines

The guidelines on urolithiasis from the European Association of Urology (EAU) and from American Urological Association (AUA) (12, 13) suggest attending a first step analysis, with no distinction between low- and high-risk patients, on all first-time stone formers and repeated in case of early recurrence after complete stone clearance intervention, early recurrence while under prevention therapy or late recurrence after long stone-free period. In this first step biochemical work-up of urine and blood and, when possible, stone composition analysis are suggested.

Once the results are obtained, the patient is assigned to a low-risk or high-risk group for stone formation based on the stone type. Only high-risk stone formers undergo specific metabolic evaluation by biochemical analysis of two consecutive 24 h-urine samples.

### 2.1 Biochemical mechanisms of stone formation

Kidney stone formation is a complex and multi-mechanistic process involving several interconnected mechanisms, influenced by chemical, physiological and anatomical conditions that act synergistically (14).

Under certain circumstances, protective mechanisms against crystallization may be altered and promote the formation of stones (e.g., matrix proteins that normally inhibit the aggregation of calcium salts). Receptors and enzyme systems such as the calcium sensing receptor (CaSR), vasopressin V2, angiotensin II and aldosterone, as well as inflammatory processes, can represent other critical factors in stone pathogenesis, transforming a protective balance into a risk for stone formation. These pathways can be altered because of genetic variants and may represent potential therapeutic targets for the development of new personalized approaches in the prevention and treatment of kidney stones (15).

### 2.2 Urine metabolic evaluation

For individuals at high risk of stone formation, the International Urolithiasis Alliance (IAU) recommends a comprehensive metabolic evaluation as well as EAU and AUA (12, 13, 16), to provide the clinician with tools to tailor nutritional and pharmacological therapies by identifying specific metabolic alterations in urine. Metabolic assessment requires 24-h urine collections (one or two samples) on a random diet, measuring at least pH, volume, calcium, uric acid, sodium, citrate, oxalate, potassium, and creatinine. The EAU and AUA recommends two consecutive samples, though spot samples may be used when 24-h collection is not feasible, particularly in children (12, 13, 17). The presence of metabolic alterations in absence of other clinical explanations should indicate a molecular analysis in order to evaluate the presence of a genetic disease.

### 2.3 Risk stratification

Currently, formal guidelines for risk stratification and genetic testing have not yet been established.

According to Gefen et al., risk indicators for genetic forms that can be used as genetic testing criteria are those associated with nephrocalcinosis and higher rates of CKD in children (younger age, low serum bicarbonate, hypophosphatemia, hypercalcemia, and developmental delay) (18).

Payne et al. Analyzed a panel of 41 genes associated with monogenic or multifactorial diseases with increased risk of nephrolithiasis in KSD patients and identified as significant predictive factors for genetic variants family history of stones (55.6%), first episode of KSD before age 18 (66.7%), recurrent stone formers, need for both medical and surgical management prior to testing (88.9%). Moreover, important indicators for genetic evaluation were 24-h urine alterations (hypercalciuria without sodium abuse, severe hyperoxaluria), or clinical suspicion for distal renal tubular acidosis (19).

Other criteria in children may include growth retardation, impaired kidney filtration, abnormal tubular function (such as acidosis, alkalosis, or electrolyte/mineral imbalances), and systemic manifestations in the eyes or bones (20).

For what concerns family history, Saha et al. showed that children carrying pathogenic variants were typically younger at symptoms onset. Additionally, only a minority of cases had family history, because 81% cases had homozygous mutations with autosomal recessive syndromes, highlighting the fact that genetic testing should be considered even when family history is absent (21). Halbritter et al. observed that recessive mutations were more frequently found among children and in congenital disease, whereas dominant conditions usually manifested later in life (22).

Therefore, the EAU and AUA criteria for identifying high-risk stone formers, which include patients with early onset urolithiasis (particularly in children and teenagers), familial stone formation, recurrent stone formation, recent stone episodes, and specific stone compositions such as brushite, uric acid, urate, or infection stones (12, 13) could also be considered amongst the inclusion criteria for genetic testing (Table 1).

**Table 1:** Findings suggestive for a genetic basis of KS.

Clinical features	Laboratory features
- Early onset of disease, particularly in children and adolescents - Family history of kidney stones - First episode of KSD before age 18 - Recurrent stone formation - Need for both medical and surgical management prior to genetic testing - Growth retardation in children - Systemic manifestations in the eyes or bones - Developmental delay	- 24-h urine alterations - Clinical suspicion of distal renal tubular acidosis - Low serum bicarbonate - Hypophosphatemia - Hypercalcemia - Impaired kidney filtration - Abnormal tubular function - Specific stone compositions such as brushite, uric acid, urate, or infection stones

## 3 Genetic testing

Several studies underline the clinical importance of genetic testing in kidney stone formers: a missed diagnosis can easily lead to kidney failure, even after a kidney transplant (23) and in the majority of the cases the kidney stones are just the first symptom of presentation of a more complex disease.

Cogal et al. in a cohort of 703 families suspected of Primary Hyperoxaluria and a cohort of 111 families suspected of Dent Disease found by targeted NGS a monogenic cause of stone formation different from the initial hypothesis in 29/285 (10.2%) and 16/59 (27.1%) respectively (24). The most common biallelically mutated gene was *CLDN16* (8 families) that together with *CLDN19* is responsible for Familial Hypomagnesemia with Hypercalciuria and Nephrocalcinosis, variants were also found in *SLC34A1* and *SLC34A3* (5 families), accounting for Hypophosphatemic/Hypercalciuric Stone Formation with Bone Defects. Among the other monogenic diseases identified there were Infantile Hypercalcemia due to 24-Hydroxylase Deficiency (*CYP24A1*), Adenine Phosphoribosyl-transferase deficiency (*APRT*), Bartter Syndrome, Distal Renal Tubular Acidosis, Autosomal Dominant Hypocalcemia (*CASR*) and Dominant Fanconi Syndrome (*HNF4A*) (24). Amlie-Wolf et al. (25) observed 28/86 pathogenic/likely pathogenic (P/LP) variants in a cohort of patients from a nephrology unit, 27/28 (96%) variants led to a change in medical management of the patient and allowed cascade testing and management of the families. In 2021 Jayasinghe et al. (26) analyzed 204 patients with a suspected monogenic cause of kidney disease with exome sequencing (WES) and a molecular diagnosis was found in 80/204 patients (34% with a P/LP variant). Among the 80 positive results, 27/80 had a confirmed diagnosis (34%), 22 had a clarified diagnosis (28%), and 31 had a reclassified diagnosis (39%), allowing a better management of the patient and a more personalized treatment (26). Andregg et al. (27) conducted a WES analysis in 901 individuals, of which 787 were kidney stone formers, 23 of them had a mendelian form of KSD and 68 had a pathogenic/likely pathogenic that not corresponded to a mendelian form but likely predisposed to nephrolithiasis. In 5 (21.5%) of the 23 mendelian form, WES established a new or corrected *a priori* diagnosis.

These are just some of the studies where a molecular diagnosis shows a high impact on the management and treatment of not just the patients themselves but also of their families.

Therefore, genetic testing in KSD could be a useful tool to predict the prognosis and the onset of other clinical features, preventing unnecessary procedures and relapses, allowing individualized treatment that are increasingly available for monogenic kidney disorders (see section 4), and offering genetic counseling for family members.

Although the availability of genetic panels for kidney stones is increasing worldwide, access varies significantly across different Countries. This contributes to the global underutilization of genetic testing in clinical practice, beside the lack of standardized guidelines (28–31).

Therefore, it is necessary to establish how to select patients eligible for genetic testing (32, 33).

### 3.1 Monogenic diseases

The use of genetic testing in KSD patients currently leads to a diagnostic yield of 12–21% in children and young adults (<25 years old) and 1–11% in adults (>25 years old) (33), where the broad ranges depend on the selected cohort and on how stringent are the criteria used for assessing the variants pathogenicity. From these studies the most frequent genes harboring pathogenic/likely pathogenic variants are those of the solute carrier families, associated with several different monogenic diseases that increase the susceptibility to KSD, followed by Primary Hyperoxaluria genes and some members of the Claudin family (6, 18, 23–28, 34–37).

There are more than 30 genes linked to monogenic diseases that imply a higher risk of stone formation (Supplementary Table S1) (38, 39). Most of the severe syndromic and congenital forms have a recessive transmission, while milder conditions are often associated with dominant pathogenetic variants. In fact, according to recent research, for some recessive conditions heterozygous individuals can no longer be considered as simple healthy carriers, because they present renal calcifications more frequently than wildtype subjects, although to a lesser extent than individuals with biallelic variants (22).

Two significant examples of monogenic diseases that cause stone formation are Dent’s disease and cystinuria.

Dent’s disease (DD) is characterized by low-molecular-weight proteinuria, hypercalciuria, nephrolithiasis and nephrocalcinosis. Two genes cause the disease: *CLCN5* (Type 1 DD) and *OCRL* (Type 2 DD). Type 2 DD may also present with extra renal symptoms, including mild cognitive impairment, hypotonia and cataracts, resulting in Lowe syndrome (40, 41).

Cystinuria is caused by mutations in *SLC3A1* and *SLC7A9* genes, leading to defective cystine transport that result in stone formation typically beginning in the first two decades of life, with higher severity in males. Cystine is an amino acid filtered by the kidneys and reabsorbed in the proximal tubule through a complex of two proteins encoded by *SLC3A1* and *SCL7A9*. Mutations in either protein impair cystine reabsorption, leading to elevated urinary excretion. Due to cystine’s poor solubility in acidic urine, this causes stone formation, primarily in the distal tubule (42). Cystinuria is well-suited for gene therapy, mainly because of the disease’s autosomal recessive nature and manageable gene sizes (*SLC3A1* 2.3 kb, *SLC7A9* 1.8 kb), also, the core of the pathogenic mechanisms is located in the proximal tubule, which is easily accessible. Studies in albino mice using CRISPR/Cas9 have shown promising results (7).

### 3.2 GWAS and WES

GWAS recognized several genetic loci and common genetic variations (present in >1% of population) associated with KSD (43–51). As an example, Hao et al. (52) conducted the largest GWAS comprising 17,969 individuals and 702,230 controls prioritized 223 unique candidate genes, some of them identified in more studies (Supplementary Table S2). Most of these genes related to kidney physiology, as *DGKH* (a component of the intracellular CaSR-signaling pathway), *CLDN14* (an integral membrane protein, component of tight junction helping decreasing the paracellular permeability to cations like calcium), *SLC34A1* (sodium and phosphate transporter), *CYP24A1* (variants lead to elevated 25-hydroxyvitamin D, increasing the reabsorption of calcium in kidney), *ALPL* (hydrolyzes extracellular inorganic pyrophosphate, which normally inhibits calcium oxalate and calcium phosphate crystallization in the urine). A significant role in nephrolithiasis predisposition is mediated by *CASR* and *TRPV5* (epithelial calcium channel transient receptor) a long-standing candidate gene for KSD and hypercalciuria. Of note are the solute carrier and the claudin families, with many different members, whose pathogenic variants are found in inherited kidney disease, and with common variants often highlighted by GWAS as susceptibility. Another example of complex pathogenic mechanism is *UMOD* (uromodulin, an inhibitor of crystallization of calcium in urine), whose pathogenic variants are responsible of medullary cystic kidney disease, but some common variants have been seen to facilitate the expression of *TRPV5* (22), therefore having a protective effect against nephrolithiasis.

Despite the large data available, the clinical use of polygenic scores for kidney diseases is limited by: (i) the scarcity of large GWAS, (ii) the low cross-ancestry transferability (Most GWAS are on European, excluding non-European population-specific variants, thus resulting in reduced predictive accuracy. E.g. the *APOL1* risk locus is not detectable in European as the risk alleles are present exclusively on African haplotypes), and (iii) variable study quality (number of subjects, quality of imputation, the extent of ancestry bias control and the control of technical artifact). Further research is needed to enhance polygenic prediction by increasing sample diversity and improving score applicability across populations.

As an example of the utility of Polygenic risk score (PRS) and Genomic-wide Polygenic Score (GPS) there is the prediction of kidney failure, that frequently rely on creatinine levels and Estimated Glomerular Filtration Rate (eGFR), which are also affected by non-renal factors such as muscle creatine production. Existing population bases-GWAS for creatinine and eGFR are more robust and statistically significant than those for CKD, due to considerably limited population available for CKD studies than the general population, where eGFR and creatinine can be examined in relation to associated genes. Consequently, PRS and GPS specific to nephrolithiasis hold significant limitations and need larger, more diverse association studies for effective application (53). Nevertheless, integrating the use of PRS and GPS with family history and biochemical parameters could help in highlighting the high-risk patients. Moreover, the interplay between monogenic variants and polygenic background should not be forgotten; indeed, the GPS for CKD was a significant predictor of kidney disease in individuals with ADPKD and COL4A-associated nephropathy variants. Among carriers of ADPKD variants, those in the top tertile of the GPS distribution had a 54-fold increased risk of CKD compared with that of non-carriers, whereas those in the bottom tertile had only a 3-fold increased risk compared to that of non-carriers (53).

## 4 New stone prevention methods

Preventive strategies are selected based on etiology, stone type, and cost-effectiveness. Main preventive measures include fluid intake (>2.5 L/day), lifestyle changes (body weight control, adequate hydration during physical activity), and dietary management (controlled intake of calcium, sodium, and animal proteins, reduced ingestion of oxalate-rich foods).

The protective effects of bioactive components are still to be explored, except for coffee which has been shown to be protective. Thiazides are indicated in people with recurrent calcium stones, and alkaline citrate for a variety of stones. Allopurinol is recommended in some forms of hyperuricosuria. Bacterial eradication and antibiotic use may be important in certain situations, but they must be closely monitored due to the possibility of antimicrobial resistance. Finally, probiotics may help in prevention, but further research is needed to prove their efficacy (54).

Recent research has identified various protein receptors and enzymes as potential therapeutic targets in urolithiasis, including calcium-sensing receptors, vasopressin V2 receptors, and angiotensin II receptors, which regulate calcium excretion, water reabsorption, and electrolyte balance. By focusing on particular pathways involved in crystal formation, aggregation, and adhesion, these receptor-targeted therapies present promising prospects for precision medicine approaches, especially in preventing stone recurrence (55).

While hyperhydration remains the foundation of prevention, understanding genetic etiology could help personalize preventive strategies.

Primary Hyperoxaluria type 1 (PH1) serves as a prime example of how understanding the genetic basis of the disease has led to targeted therapy directly addressing the pathogenesis. PH1 is caused by pathogenic variants in the *AGXT* gene, encoding for the liver enzyme alanine glyoxylate aminotransferase (AGT), leading to excess oxalate production (56). Pyridoxine acts as a cofactor for AGT enzyme, increasing its activity and reducing oxalate production in specific *AGXT* variants (57). Lumasiran is a RNA interference therapeutic agent administered subcutaneously and directed at the liver. In patients with PH1, Lumasiran reduces hepatic oxalate production and increases glycolate concentrations by degrading the messenger RNA that encodes AGT (8). Other examples are the specific therapy in patients with CYP24A1 gene variants comprises azole drugs (e.g., ketoconazole and fluconazole), which inhibit cytochrome P450 enzymes, leading to normalization of biochemical parameters (58, 59). Another approach is to induce CYP3A4 activity with the drug rifampicin, providing an alternative method for the inactivation of active vitamin D metabolites (60). For SLC34A1 gene variants, the specific therapy consists of oral phosphorus supplementation, which leads to rapid correction of hypophosphatemia, normalization of calcium metabolism, and significant clinical improvement, as demonstrated by weight gain in patients. Sodium chloride supplementation may also be necessary to correct hypercalcemia in patients who also present with polyuria and polydipsia (61). For Cystinuria, cystine-binding drugs such as Tiopronin and D-penicillamine, are effective in reducing free urine cystine levels (62). Moreover, l-Cystine diamides, inhibitors of l-cystine crystallization (63), and *α*-Lipoic acid that inhibits cystine stone formation (64) have shown great promise in the treatment of Cystinuria but are still under evaluation. Early diagnosis of Adenine phosphoribosyltransferase (APRT) deficiency allows for treatment with allopurinol, which inhibits xanthine oxidase and reduces 2,8-DHA formation, potentially preventing, arresting, or even reversing kidney failure in both native and transplanted kidneys (65). In clinical trials Febuxostat showed better results than allopurinol in reducing DHA excretion in the prescribed doses (66).

Thus, early genetic diagnosis can enable targeted therapies, sparing kidney-liver transplantation to the patients and long-term complication (67).

## 5 Conclusion

**Figure 1:**
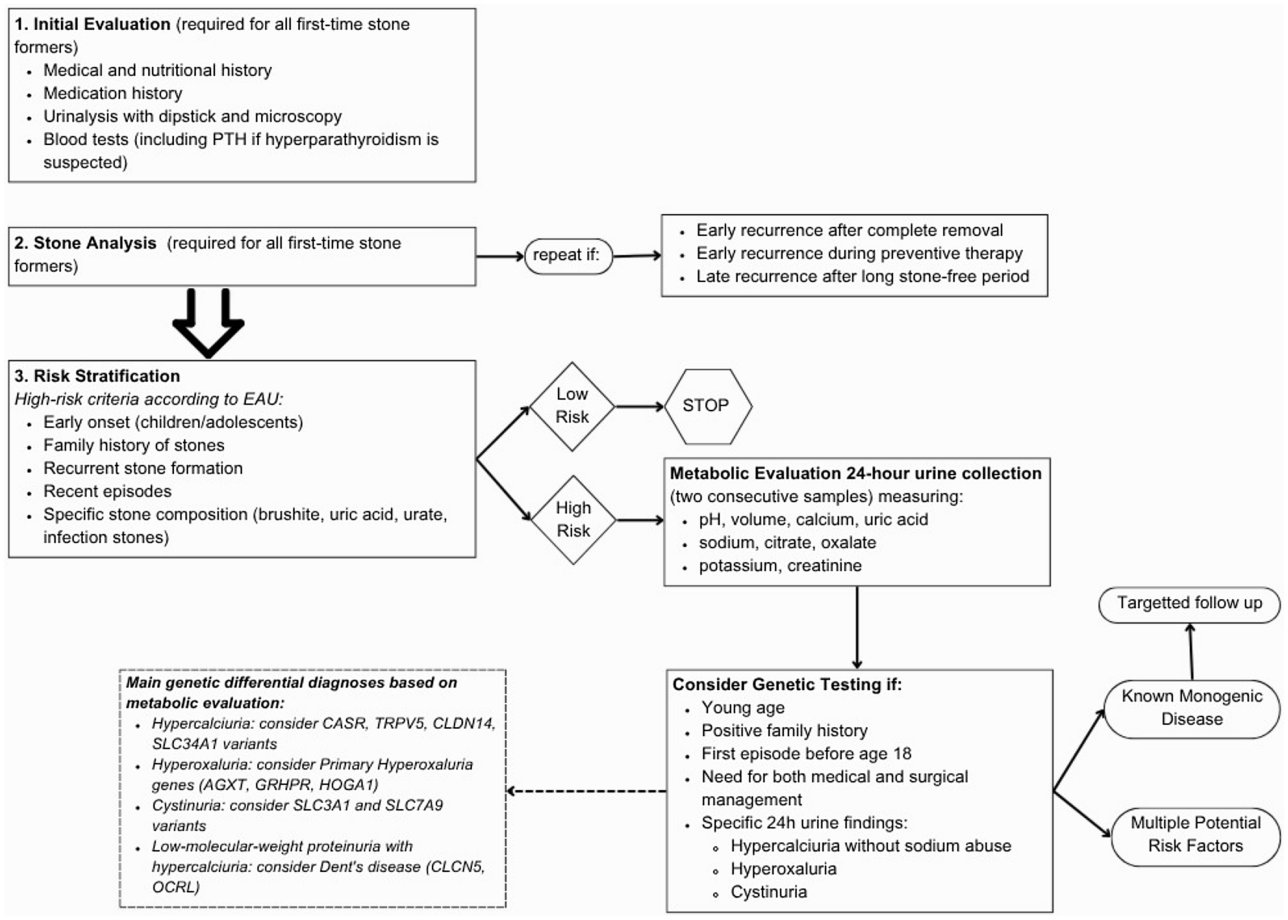
Flow chart to guide genetic testing; see Supplementary Table S1 for description of the diseases associated to each gene (67).

Despite the KSD genetic predisposition is not yet fully understood, the present data are sufficient to recommend genetic testing of a stone-gene panel in high-risk KSD patients. In conclusion, Red Flags to recognize genetic forms in KSD patients can be: recurrent nephrolithiasis, first episode of KSD before the age of 18 years old, nephrocalcinosis, family history of stones, 24-h urine alterations, impaired kidney filtration, abnormal tubular function and syndromic conditions (Figure 1). The clinical utility of genetic diagnosis goes far beyond therapeutic applications only. It includes pre-symptomatic identification of monogenic forms before the development of irreversible renal damage, support for clinical management through more targeted monitoring, detection of recessive disorder heterozygous carriers who may be at increased risk, and utilization of tailored preventive interventions based on the individual genetic pattern. Furthermore, it allows for counseling of high-risk relatives, offers the possibility of prenatal diagnosis in the most severe cases, and directs future research toward new treatments this broader vision of the utility of genetic diagnostics highlights the importance of integrating genetic testing into the diagnostic workup of patients with recurrent renal lithiasis or with clinical features suggestive of hereditary forms.

Undoubtedly, genetics will not be the only key to better understanding. Indeed, multi-omic studies have consistently shown that kidney stone patients exhibit gut and urinary dysbiosis with reduced oxalate-degrading bacteria (e.g., *O. formigenes*, *Lactobacillus*, *Bifidobacterium*) and enrichment of pathogens (e.g., *Streptococcus*, *E. coli*), alongside metabolic alterations involving tryptophan, fatty acids, and oxalic acid (68–70). These changes correlate with inflammation, oxidative stress, and urinary metabolite shifts (e.g., pyroglutamic acid, FAHFA), supporting the need of biomarker discovery and microbiome-targeted therapies such as probiotics and FMT (Fecal Microbiota Transplantation) (68, 69).
