# Supplementary material for: Genetics of kidney stones and the role of genetic testing in prevention: a guide for urologists
Source: Front Med (Lausanne). 2025 Jul 25;12:1631281. doi: 10.3389/fmed.2025.1631281 (PMC12331670; doi:10.3389/fmed.2025.1631281)
Supplement: Supplementary file 1 [file Table_1.docx]

***Supplementary Material***

**Supplementary Table 1.** Most frequent monogenic disease associated with KS, with corresponding genes and type of stone and treatment available (67)

| **Gene** | **Protein** | **Disease** | **Type of Stones** | **New stone prevention methods** |
| --- | --- | --- | --- | --- |
| **Associated with hypercalciuria** | | | | |
| ADCY10 | Adenylate cyclase type 10 | Idiopathic Hypercalciuria, AD | Calcium oxalate and calcium phosphate stones |  |
| VDR | Vitamin D3 receptor | Idiopathic Hypercalciuria, AD | Calcium oxalate and calcium phosphate stones |  |
| CASR | Extracellular calcium-sensing receptor | Hypocalcemia Type 1, AD; Bartter Syndrome Type 5, AD | Calcium oxalate and calcium phosphate stones | CaSR antagonists (calcilytics) are under development, CaSR agonists (calcimimetics) |
| GNA11 | Guanine nucleotide-binding protein subunit alpha-11 | Hypocalcemia Type 2, AD | Calcium oxalate and calcium phosphate stones |  |
| NKCC2 | Solute carrier family 12 member 1 | Bartter Syndrome Type 1, AR | Calcium oxalate and calcium phosphate stones |  |
| ROMK | ATP-sensitive inward rectifier potassium channel 1 | Bartter Syndrome Type 2, AR | Calcium oxalate and calcium phosphate stones |  |
| CLCNKB | Chloride channel protein ClC-Kb | Bartter Syndrome Type 3, AR | Calcium oxalate and calcium phosphate stones | Mineral supplementation, Indomethacin |
| BSND | Barttin | Bartter Syndrome Type 4, AR | Calcium oxalate and calcium phosphate stones |  |
| CLCN5 | H(+)/Cl(-) exchange transporter 5 | Bartter Syndrome Type 6, XLR, Dent Disease Type 1, XLR | Calcium oxalate and calcium phosphate stones |  |
| OCRL | Inositol polyphosphate 5-phosphatase OCRL | Dent Disease Type 2, XLR | Calcium oxalate and calcium phosphate stones |  |
| SLC4A1 | Band 3 anion transport protein | Distal Renal Tubular Acidosis, AD | Calcium phosphate stones | Alkali therapy |
| ATP6V1B1 | V-type proton ATPase subunit B, kidney isoform | Distal Renal Tubular Acidosis, AR | Calcium phosphate stones | Alkali therapy |
| ATP6V0A4 | V-type proton ATPase 116 kDa subunit a 4 | Distal Renal Tubular Acidosis, AR | Calcium phosphate stones | Alkali therapy |
| CA2 | Carbonic anhydrase 2 | Distal Renal Tubular Acidosis, AR | Calcium phosphate stones |  |
| FOXI1 | Forkhead box protein I1 | Distal Renal Tubular Acidosis, AR | Calcium phosphate stones |  |
| WDR72 | WD repeat-containing protein 72 | Distal Renal Tubular Acidosis, AR | Calcium phosphate stones |  |
| SLC34A3 | Sodium-dependent phosphate transport protein 2C | Hereditary Hypophosphatemic Rickets with Hypercalciuria, AR | Calcium oxalate and calcium phosphate stones |  |
| CLDN16 | Claudin-16 | Familial Hypomagnesemia with Hypercalciuria and Nephrocalcinosis, AR | Calcium oxalate and calcium phosphate stones |  |
| CYP24A1 | 1,25-dihydroxyvitamin D(3) 24-hydroxylase, mitochondrial | Infantile Hypercalcemia Type 1, AR | Calcium oxalate and calcium phosphate stones | Azole drugs, rifampicin |
| SLC34A1 | Sodium-dependent phosphate transport protein 2A | Infantile Hypercalcemia Type 2, AR | Calcium oxalate and calcium phosphate stones | Oral phosphorus supplementation, Sodium chloride supplementation |
| **Not associated with hypercalciuria** | | | | |
| AGXT | Alanine-glyoxylate aminotransferase | Primary Hyperoxaluria, Type 1, AR | Calcium oxalate stones | Lumasiran, Pyridoxine |
| GRHPR | Glyoxylate reductase/hydroxypyruvate reductase | Primary Hyperoxaluria, Type 2, AR | Calcium oxalate stones |  |
| HOGA1 | 4-hydroxy-2-oxoglutarate aldolase, mitochondrial | Primary Hyperoxaluria, Type 3, AR | Calcium oxalate stones |  |
| SLC3A1 | Amino acid transporter heavy chain SLC3A1 | Cystinuria, AD, AR | Cystine stones | Tiopronin, D-penicillamine, l-Cystine diamides, α-Lipoic acid |
| SLC7A9 | b(0,+)-type amino acid transporter 1 | Cystinuria, AD, AR | Cystine stones | Tiopronin, D-penicillamine, l-Cystine diamides, α-Lipoic acid |
| HPRT1 | Hypoxanthine-guanine phosphoribosyltransferase | Hereditary Hyperuricosuria, XLR | Uric acid stones |  |
| PRPS1 | Ribose-phosphate pyrophosphokinase 1 | Hereditary Hyperuricosuria, XLR | Uric acid stones | Xanthine oxidoreductase inhibitors, Purine restriction |
| SLC2A9 | Solute carrier family 2, facilitated glucose transporter member 9 | Hereditary Hyperuricosuria, XLR | Uric acid stones | Xanthine oxidoreductase inhibitors |
| XDH | Xanthine dehydrogenase/oxidase | Hereditary Xanthinuria Type 1, AR | Xanthine stones | Purine restriction |
| MOCOS | Molybdenum cofactor sulfurase | Hereditary Xanthinuria Type 2, AR | Xanthine stones |  |
| APRT | Adenine phosphoribosyltransferase | Adenine Phosphoribosyltransferase Deficiency, AR | 2,8-dihydroxyadenine (DHA) stones | Allopurinol, Febuxostat |

**Supplementary Table 2.** Genes most frequently identified in multiple recent GWAS, with corresponding study counts.
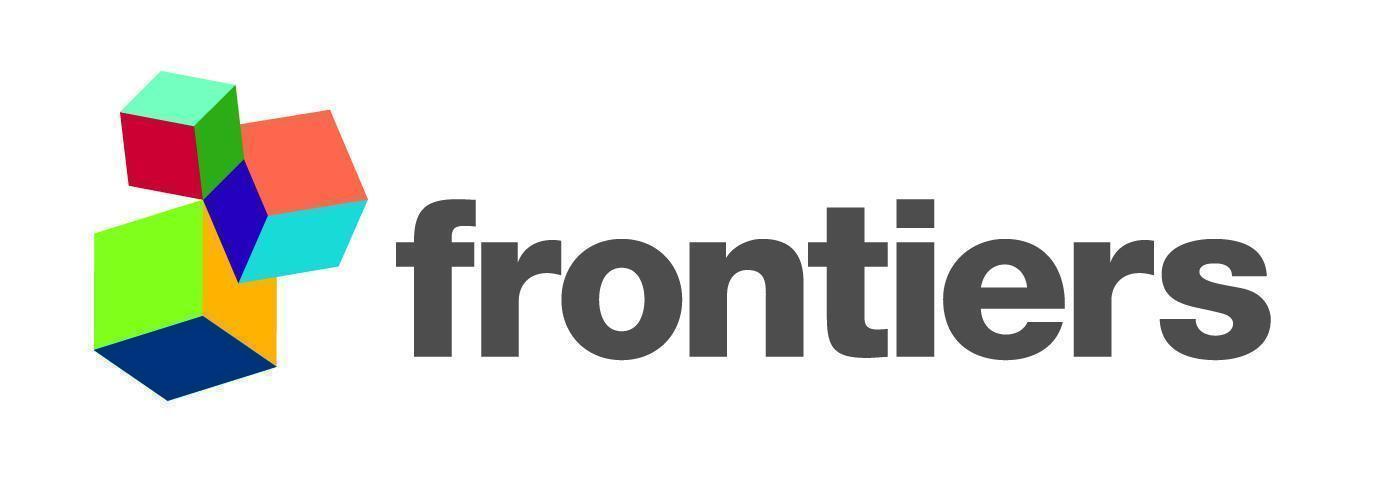


| **Gene** | **Protein** | **Papers citing gene (n)** | **References** |
| --- | --- | --- | --- |
| DGKH | Diacylglycerol Kinase Eta - involved in cellular signaling and lipid metabolism | 6 | (46, 48-52) |
| CLDN14 | Claudin 14 - integral membrane protein component of tight junctions in the kidney | 5 | (43, 45, 48, 49, 52) |
| SLC34A1 | Sodium-Dependent Phosphate Transport Protein 2A - involved in phosphate reabsorption in the kidney | 5 | (45, 46, 49, 50, 52) |
| CYP24A1 | 1,25-dihydroxyvitamin D3 24-hydroxylase - involved in vitamin D metabolism | 4 | (48-50, 52) |
| UMOD | Uromodulin - involved in kidney function | 4 | (44, 49, 50, 52) |
| ALPL | Alkaline Phosphatase - enzyme that removes phosphate groups from molecules | 3 | (45, 49, 52) |
| GCKR | Glucokinase Regulatory Protein - regulates glucose metabolism | 3 | (49, 50, 52) |
| PDILT | Protein Disulfide Isomerase-Like Testis - involved in protein folding | 3 | (50, 51, 52) |
| SHROOM3 | Shroom Family Member 3 - regulates cell shape and tissue morphogenesis | 3 | (44, 50, 52) |
| AQP1 | Aquaporin 1 - water channel protein facilitating water transport across cell membranes | 2 | (46, 52) |
| BCAS3 | Breast Carcinoma Amplified Sequence 3 - involved in cell migration and tissue development | 2 | (50, 52) |
| CASR | Calcium Sensing Receptor - involved in calcium homeostasis | 2 | (45, 52) |
| DGKD | Diacylglycerol Kinase Delta - cellular signaling pathway protein | 2 | (48, 52) |
| FAM188B | Family with Sequence Similarity 188 Member B - involved in proteolysys | 2 | (46, 49) |
| SPATA5L1 | Spermatogenesis-Associated Protein 5-Like 1 - hydrolyze ATP to induce changes in target substrates | 2 | (44, 50) |
| KCNK5 | Potassium Channel K5 - potassium ion transporter | 2 | (49, 51) |
| LRP2 | LDL Receptor Related Protein 2 - involved in receptor-mediated endocytosis | 2 | (47, 50) |
| NFATC1 | Nuclear Factor of Activated T Cells 1 - transcription factor in immune response | 2 | (50, 51) |
| SLC22A2 | Solute Carrier Family 22 Member 2 - organic cation transporter | 2 | (50, 52) |
| TFAP2B | Transcription Factor AP-2 Beta - Involved in development and differentiation | 2 | (49, 52) |
| TRPV5 | Transient Receptor Potential Cation Channel V5 - Calcium channel involved in calcium reabsorption | 2 | (45, 52) |
| WDR72 | WD Repeat Domain 72 - involved in enamel formation and mineralization | 2 | (48, 50) |
| ABCG2 | ATP Binding Cassette Subfamily G Member 2 - membrane transporter protein | 1 | (51) |
| AP003068.2 | CAPN1 antisense RNA 1 | 1 | (51) |
| BCR | Breakpoint Cluster Region Protein - GTPase-activating protein | 1 | (48) |
| CLDN10 | Claudin 10 - tight junction protein | 1 | (52) |
| COL4A3 | Collagen Type IV Alpha 3 Chain - basement membrane collagen | 1 | (47) |
| CUBN | Cubilin - receptor involved in vitamin and protein absorption | 1 | (47) |
| EPB41L2 | Erythrocyte Membrane Protein Band 4.1 Like 2 - cytoskeletal protein | 1 | (49) |
| FTO | Alpha-Ketoglutarate-Dependent Dioxygenase - involved in nucleic acid demethylation | 1 | (49) |
| GIPC1 | GIPC PDZ Domain Containing Family Member 1 **-** regulates cell surface receptor expression and trafficking | 1 | (48) |
| HDAC4 | Histone Deacetylase 4 - transcriptional regulator | 1 | (51) |
| HIBADH | 3-Hydroxyisobutyrate Dehydrogenase - enzyme of amino acid metabolism | 1 | (52) |
| LRRC37A2 | Leucine Rich Repeat Containing 37 Member A2 - involved in cellular signaling and transmembrane transport | 1 | (52) |
| PCDH15 | Protocadherin 15 - protein involved in hearing and balance | 1 | (51) |
| PLEKHM1 | Pleckstrin Homology Domain Containing M1 - involved in bone resorption | 1 | (52) |
| PM20D1 | Peptidase M20 Domain Containing 1 - metabolic enzyme | 1 | (52) |
| PRELID1 | PRELI Domain Containing 1 - involved in phospholipid transfer | 1 | (52) |
| RGS14 | Regulator of G Protein Signaling 14 - Regulator of G protein signaling | 1 | (52) |
| RN7SKP27 | RNA, 7SK Small Nuclear Pseudogene 27 - Non-coding RNA | 1 | (51) |
| SCNN1B | Sodium Channel Epithelial 1 Beta Subunit - involved in sodium transport | 1 | (52) |
| SLC12A1 | Solute Carrier Family 12 Member 1 - sodium/potassium/chloride transporter | 1 | (52) |
| SLC30A10 | Solute Carrier Family 30 Member 10 - zinc transporter | 1 | (52) |
| SLC39A11 | Solute Carrier Family 39 Member 11 - metal ion transporter | 1 | (52) |
| SLC5A2 | Sodium/Glucose Cotransporter 2 - glucose transporter | 1 | (47) |
